# Teenage Curiosity: Magnetic Attraction Gone Wrong

**DOI:** 10.5811/cpcem.2019.5.42879

**Published:** 2019-07-01

**Authors:** Matthew Hysell, Sarah E. Harris-Kober

**Affiliations:** Michigan State University College of Osteopathic Medicine, Spectrum Health-Lakeland, Department of Emergency Medicine, St. Joseph, Michigan

## Abstract

A 13-year-old male presented with suprapubic pain, hesitancy, and dysuria beginning seven hours prior to arrival. After initial evasiveness, the patient admitted to inserting small, magnetic ball bearings into his penis. Vital signs and physical exam were unremarkable aside from mild suprapubic tenderness to palpation. Pelvic radiograph demonstrated about 45 radiopaque beads within the urethra and bladder. While urethral foreign body (FB) is an uncommon diagnosis, it is essential to identify quickly as lifelong complications can arise. Magnetic FBs are particularly concerning due to possible ischemia from compression injury and difficulty of removal. Safety concerns led to temporary market removal of neodymium magnetic toys, but sales resumed in 2016.

## CASE PRESENTATION

A healthy 13-year-old male presented with suprapubic pain, hesitancy, and dysuria beginning seven hours prior to arrival. After initial hesitancy, the patient admitted to inserting small, magnetic ball bearings into his penis over the prior month, stating “I never lost any until today.” Vital signs and physical exam were largely unremarkable aside from mild suprapubic tenderness to palpation. Urinalysis demonstrated 31–50 leukocytes per high powered field (HPF) and too numerous to count red blood cells/HPF. Complete blood count and comprehensive metabolic panels were within normal limits.

**Image 1:**
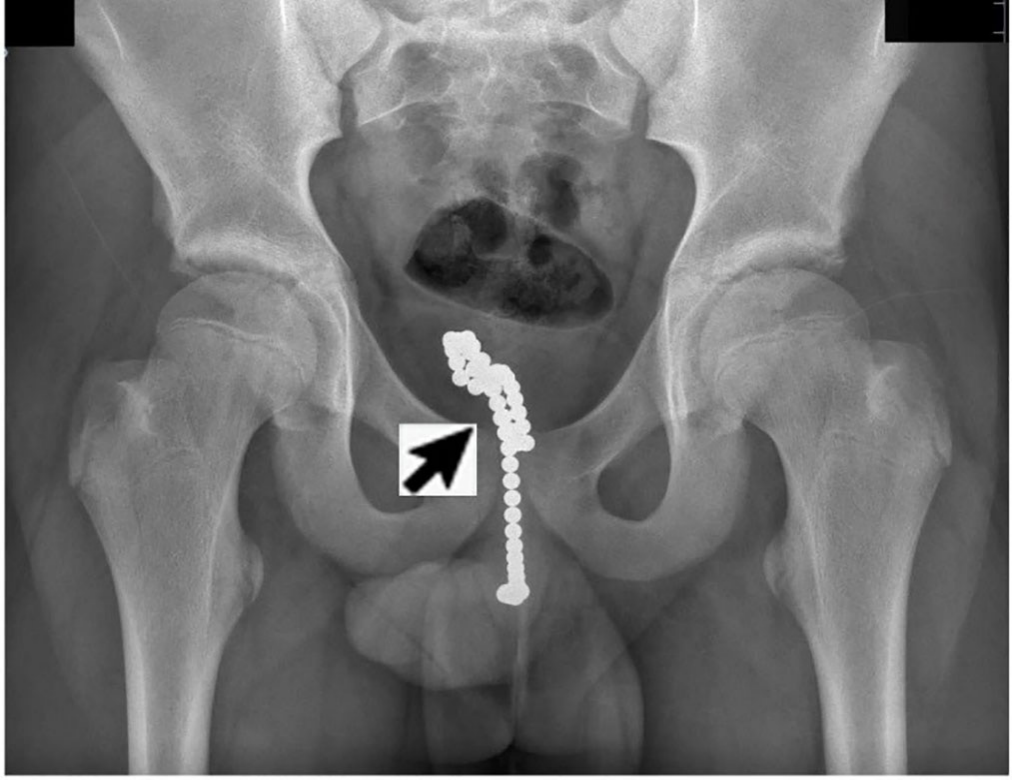
An anterior posterior plain film radiograph of the pelvis showing magnetic ball bearings in bladder and urethra.

**Image 2:**
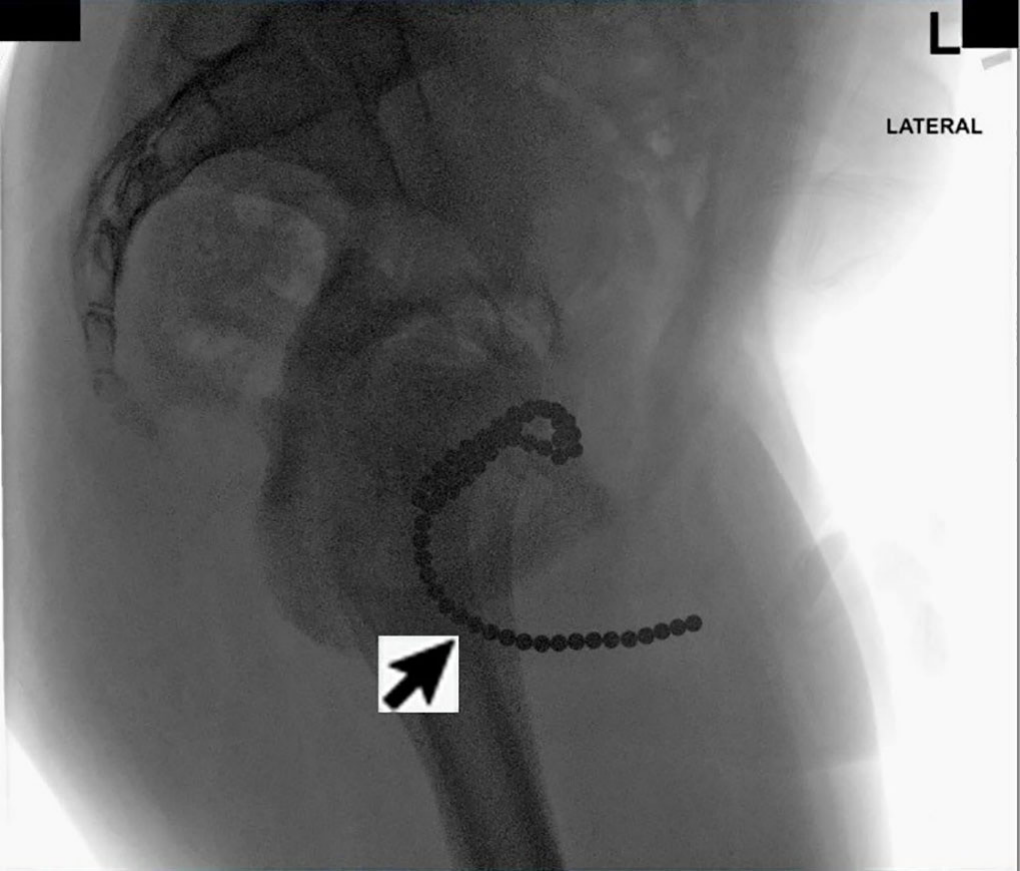
A lateral plain film radiograph of the pelvis demonstrating magnetic ball bearings in bladder and urethra.

Due to concern for retained foreign body (FB), pelvic radiograph was obtained. This demonstrated about 45 small, round, radiopaque beads within the urethra and bladder consistent with those brought in by the boy’s father (Images 1 and 2). Post-void residual bladder scan revealed 69 milliliters of urine prior to patient reporting complete bladder emptying in the ED. Outpatient urologic surgery was arranged for the next day with prescription for prophylactic cephalexin. Our community urologist was unable to remove the magnetic bearings, and the patient was referred to pediatric urology at a tertiary location.

## DISCUSSION

Diagnosis of urethral FBs is challenging given patients’ hesitance to report insertion of a FB. Delayed presentation and diagnosis increases complication rates.^1^

Polyembolokoilamania, or insertion of an object into a bodily orifice for sexual gratification, is the most common cause of urethral FBs.^2^ Other motivations for insertion include curiosity or as a voiding aide.^3^

Urethral FB presentation is similar to infection or stricture. Complications include chronic cystitis, hydronephrosis, secondary stone formation, and renal failure.^1^,^4^ It is essential, especially in populations with low rates of voiding difficulty or infection, such as children and teenagers, to consider urethral FB.

Magnetic FBs are particularly concerning due to possible urethral or bladder wall compression resulting in ischemia.^3^ Removal of these objects is difficult due to their magnetic adhesion. While neodymium magnetic toys were temporarily removed from the market, retail sales resumed in 2016 and they continue to pose a risk in the community.

**CPC-EM Capsule**

What do we already know about this clinical entity?

The most common cause of urethral foreign body (FB) is polyembolokoilamania. This often leads to delayed presentation and failure to report insertion.

What is the major impact of the image(s)?

Magnet urethral FBs are particularly concerning due to possible serious complications. Neodymium beads were temporarily removed from the market but returned in 2016.

How might this improve emergency medicine practice?

It is important to consider urethral FB in populations with low risk of voiding difficulty. Early detection is essential as serious, life-long complications can occur.
